# Squint—Its Causes, Pathology and Treatment

**Published:** 1903-10

**Authors:** 


Squint—Its Causes, Pathology and Treatment. By Claud Worth, F. R. C. S. Philadelphia: P. Blakiston's Son & Co. 1903.

In these days of much bookmaking, a writer who has original ideas and who presents them in a clear forceful way so as to carry conviction, is entitled to more than a perfunctory word of commendation. Such a writer is Claud Worth, whose studies on "Squint" have been most careful, and whose conclusions are revolutionary. Only the first chapter deals with a common subject in a stereotyped way. The remainder of the book is replete with careful observations, valuable suggestions and logical conclusions.

The most important of his studies is on the fusion function,—the causes of the amblyopia, which develops in the squinting eye, and the method of training, by which the sight may be preserved, if only the visual exercises prescribed are undertaken at a sufficiently early age. The writer makes the assertion not generally recognised by ophthalmologists that the fusion function is attained by the sixth or seventh year, and that the correction of the amblyopia depends upon treatment being instituted before this age has been reached. Children of two years, showing refractive errors and tendencies toward convergence, are given glasses (which can be accurately prescribed by the aid of the shadow test), and the fusion function, unless totally absent, developed by means of exercises with the amblyoscope. Operative measures are in this way, in many cases, altogether avoided, and when imperative, vastly better optical and cosmetic results are obtained if preceded by visual correction and training. The author follows Landolt in advancing a weak antagonist, rather than by excessive tenotomy of the stronger muscle, and a practical test of his method demonstrates it to be simple and efficient. The book, in a word, is worthy of a careful study, quite as much by the progressive general practitioner as by the specialist, as the future welfare of many eyes will depend upon the early and intelligent advice of the family physician. F. P. L.

